# Attitudes towards living organ donation: a cross-sectional survey study

**DOI:** 10.3389/fpubh.2025.1552393

**Published:** 2025-03-26

**Authors:** Sydney Naibauer, William T. Branagan, Stephanie Lehto, Nicole Reynolds, Susan Mikulich-Gilbertson, Chloe E. Page, Rachel A. Davis

**Affiliations:** ^1^Department of Psychiatry, University of Colorado Anschutz Medical Center, Aurora, CO, United States; ^2^Colorado Center for Transplantation Care, Research and Education (CCTCARE), University of Colorado Anschutz Medical Center, Aurora, CO, United States; ^3^Department of Neurosurgery, University of Colorado Anschutz Medical Center, Aurora, CO, United States

**Keywords:** living organ donation, living liver donation, living kidney donation, altruism, non-directed living donation, organ donation, altruistic living donor

## Abstract

**Objective:**

Only 18% of kidneys and livers transplanted in the United States come from living donors, and increasing rates of living organ donation could help decrease the critical organ deficit. Non-directed living donation is even less common, with only 1.4% of kidney and liver transplants coming from anonymous donors (1). This study aimed to determine which factors are considered more motivating and more discouraging to living organ donation, how characteristics of potential recipients affect willingness to consider living liver donation, and whether there are any associations related to a person’s willingness to consider non-directed living organ donation.

**Method:**

A cross-sectional survey was distributed in-person on a large medical campus, and participation was incentivized with the opportunity to spin a prize wheel. In addition to participant characteristics, the survey queried awareness of directed and non-directed living donation, whether or not the participant would consider directed donation and non-directed donation, motivating and discouraging factors to living donation, vignettes to assess willingness to donate to recipients with different characteristics, and an altruism personality inventory. An optional interpersonal reactivity index was included as well.

**Results:**

Three hundred twenty-six participants scanned a QR code to take the survey. Most participants (299 of 318, 94%) were aware of living donation. Participants who said yes to considering non-directed living donation (67 of 305, 22%) had significantly higher altruism scores than those who said no (123 of 305, 40%). Willingness to consider living liver donation varied based on recipient characteristics, with participants reporting they would be more willing to donate to a recipient with an immune disorder over alcohol-related liver disease, an infant over an adult, a relative over a nonrelative, and a sibling with alcohol-related liver disease over a nonrelative with alcohol-related liver disease.

**Conclusion:**

The most motivating factors for considering living donation were having a child recipient, helping someone in need, high transplant center success rate, and helping a family member or friend. The most discouraging factors were uncompensated expenses, difficulty of surgery recovery, risk of surgery, and length of recovery. Participants were less willing to donate to adults, strangers, and recipients with alcohol-related liver disease.

## 1 Introduction

In the United States, there is a critical shortage of organs available for transplantation. As of February 2025, there were 104,298 candidates on the transplant waiting list (99,352 waiting for a kidney or liver). In 2024, 39,217 candidates received kidney or liver transplants, and 10,057 kidney or liver candidates were removed from the waiting list, either due to death or becoming too sick to transplant (1). Living organ donation offers the benefit of reduced wait times for a transplant and improved outcomes for patients waiting for an organ (2). Living donors are individuals who donate an organ while they are alive, in contrast to deceased donation following their death. The two most commonly donated organs from living donors are the kidney and liver (1). Of the 39,217 transplants in 2024, 18% (7,022) were from living donors (6,418 kidneys and 604 livers). This is fewer than the 7,397 living organ donations performed in 2019 (1). The COVID-19 pandemic further decreased living donor rates, and though those rates may be recovering (3), living organ donation remains uncommon.

Most living organ donations are directed donations, which is when the donor chooses to donate to a specific person with whom they have a relationship (1). Some transplant centers in the United States also perform non-directed organ donation, where the donation is made to a person unknown to the donor. People are generally more open to directed donation than non-directed, or anonymous, donation (4). In 2024, only 1.4% of kidney (455) and liver (98) transplants were from non-directed donors (1).

### 1.1 Motivations for living donation

Living organ donation, whether directed or non-directed, is generally viewed as an altruistic act. The desire to save a life and to do something good are common motivators (5). A study of 14 focus groups of living donors’ evaluation experiences found donors become emotionally invested and prioritize the recipient’s health over the potential risks to themselves (6). Some authors have proposed alternative reasons for donation beyond altruism, including a family member’s desire to alleviate caregiving responsibilities (7).

Cultural factors also impact rates of living organ donation. Western societies have tended to focus on promoting deceased donation, whereas Eastern/Asian societies promote living donation, largely due to strong cultural and religious beliefs that the body should be kept intact for burial and the afterlife (8). In the East, over 90% of liver transplants are from living donors (9).

### 1.2 Barriers to living donation

While someone may be motivated to become a living donor, there are several barriers prospective donors face which may prevent donation. For example, one multicenter study of living donors found that 89% of donors had incurred a net financial loss, with direct and indirect costs including ground transportation, health care, medications, and lost wages. One third of donors experienced a loss of more than $2,500 (10). Other reported barriers include fear of surgical complications, gaps in knowledge about living donation, risks to the donor, uncertainty about organ donation and processes, cultural barriers, recipient indebtedness, and lack of trust in healthcare (4).

Knowledge about living donation procedures, laws, and other relevant factors may contribute to or impact the willingness of living organ donation (11). Public opinions are more supportive of living donation to a child, spouse, or sibling than they are of a friend or anonymous recipient (4). However, donation to a stranger through paired kidney exchange, where a donor who is not a match for an intended recipient is “exchanged” with another donor who is a match (and vice-versa), may be more accepted as it is considered reciprocal and ultimately benefits a donor’s intended recipient (4).

Most literature on the motivations and barriers to living organ donation focuses on kidney donation and not living liver donation (12, 13). This study examines recipient characteristics such as age, relationship to the participant, and reason for needing a transplant to determine if these characteristics affect participants’ willingness to consider living liver donation. This study also aimed to determine which factors are perceived as more motivating and which are perceived as more discouraging to living organ donation and potential associations with willingness to donate. This knowledge can guide future interventions to increase living organ donation, which will help address the critical shortage of organs available for transplantation.

## 2 Materials and methods

### 2.1 Design

This quantitative and qualitative survey research utilized a single site, cross-sectional design on a medical campus in Colorado (United States). The study received Institutional Review Board (IRB) (24–1,299) approval at the University of Colorado Anschutz. Given the minimal risk and anonymous nature of the primary survey, this research qualified for exempt status. Consent information was provided at the beginning of the survey, and continuing the survey confirmed participant consent.

### 2.2 Setting

This in-person survey was completed on the Anschutz Medial Campus, a public education, clinical, and research facility, serving approximately 4,500 health professional and graduate students and employing approximately 10,000 faculty and staff. In addition to education and research buildings, Anschutz Medical Campus hosts many outpatient clinics and a large metropolitan hospital.

### 2.3 Population

Participants were not asked to define their role on campus, but common visitors and occupants to the survey administration sites include health professional students, clinicians, researchers, administrative staff, other staff, and patients.

### 2.4 Sampling

The only inclusion criterion was that participants be 18 years of age or older. Participants were recruited in-person at a table by a coffee shop in combined research/clinical/administrative building on the Anschutz Campus, or by food trucks at a central campus location. Participants were either drawn spontaneously to the large prize wheel and table with prizes or were approached by study personnel as they walked by or sat at nearby tables. Potential participants were told that the survey is on living organ donation and does not collect personal identifying information. In addition to collecting the data described below, the survey provided educational information about living organ donation and statistics on the organ shortage in the United States.

### 2.5 Survey

Study data were collected and managed using the cloud-based survey platform, Qualtrics, hosted at the study site (University of Colorado Anschutz). One participant completed the survey on a tablet provided by the study personnel. All other participants completed the survey on a personal device by scanning a QR code. No identifying information was collected on the primary survey. At the end of the survey, participants were directed to a new link with a question about whether they would be interested in speaking with a living donor. They were informed that, if they chose to provide contact information, it would not be linked to their previous survey responses, so their responses would remain anonymous. This information was only accessed by the first and senior authors (SN, RD). Once the participants completed the survey, they could spin a prize wheel. The wheel was arranged such that most participants won a vinyl “give life” liver or kidney sticker (value of approximately $1). Bigger prizes included plush organs, donation-related T-shirts, organ pins, $5 coffee gift cards, and blue or green insulated tumblers. The maximum prize value was $35.

#### 2.5.1 Participant characteristics

The survey asked participant’s age range, level of education, gender, income level, and race/ethnicity.

#### 2.5.2 Altruism facet of the HEXACO-PI-R

The 200-item full-length HEXACO Personality Inventory Revised assesses six broad personality factors (Honesty-humility, Emotionality, Extraversion, Agreeableness, Conscientiousness, and Openness to experience), each with four narrower personality characteristics, or facets (14, 15). A 25^th^ facet, “altruism,” combines four items from the honesty-humility, emotionality, and agreeableness factors. The altruism facet measures attitudes and concepts, rather than behaviors.

#### 2.5.3 General questions

Participants were asked about their awareness of living organ donation, whether or not someone was or had been a living donor (if affirmative, followed by a qualitative question about reasons for donating), willingness to consider participating in directed and non-directed living organ donation, a qualitative question about why they might or might not consider donating, and willingness to consider becoming a living donor in the future.

#### 2.5.4 Motivating factors to consider becoming a living donor

Participants were provided 19 potential motivating factors and asked to rank each on a Likert scale with 1 being “not at all motivating” and 5 being “extremely motivating” (Table 1).

**Table 1:** Motivating factors to consider becoming a living donor.

Factor number	Factor
1	Shorter surgery recovery time
2	Shorter hospital stay
3	Age of organ recipient-child
4	Age of organ recipient-adult
5	Taking time off work
6	High success rate of transplant center
7	Cultural or religious factors
8
Comfort with medical procedures	9	Scar from surgery
10	Shortage of organs available for transplant
11	Recognition for being a donor
12	Financial compensation for any lost income or expenses
13	Financial compensation above and beyond lost income or expenses
14	The way my friends and/or family would react
15	The way society might perceive me
16	Getting to meet the recipient after donation
17	The opportunity to help someone in need and potentially save a life
18	The opportunity to do something unique that very few people in the world get to do
19	Need of a family member or friend

Participants were asked to rank 19 potentially motivating factors on a Likert scale with 1 being “not at all motivating” and 5 being “extremely motivating.”

#### 2.5.5 Discouraging factors to consider becoming a living donor

Participants were provided 14 potential discouraging factors and asked to rank each on a Likert scale with 1 being “not at all discouraging” and 5 being “extremely discouraging” (Table 2).

**Table 2:** Discouraging factors to consider becoming a living donor.

Factor number	Factor
1	Risk of surgery
2	Length of recovery from surgery
3	Difficulty of recovery from surgery
4	Age of organ recipient-child
5	Age of organ recipient-adult
6	Taking time off work
7	Uncompensated expenses related to donating
8	Cultural or religious factors
9	Discomfort with medical procedures
10	Scar from surgery
11	Recognition for being a donor
12	The way my family and/or friends would react
13	The way society might perceive me
14	Getting to meet the recipient after donation

Participants asked to rank 14 potential discouraging factors on a Likert scale with 1 being “not at all discouraging” and 5 being “extremely discouraging.”

#### 2.5.6 Vignettes

Participants were provided six different scenarios and asked to rate their willingness to donate part of their liver on a scale from 1 (very unwilling to donate) to 5 (very willing to donate) for: (1) an unrelated 6-month-old infant with a congenital liver disease, (2) their own 6-month-old child with a congenital liver disease, (3) an unrelated adult with liver disease due to an immune disorder, (4) an unrelated adult with alcohol related liver disease, (5) their own adult sibling with liver disease due to an immune disorder, and (6) their own adult sibling with alcohol related liver disease (Table 3).

**Table 3:** Participants were asked to rate their willingness to donate part of their liver for six different scenarios, with 1 being “very unwilling to donate” and 5 being “very willing to donate.

Scenario number	Scenario
1	Your 6-month-old child with a congenital (born with) liver disease (biliary atresia) requires a liver transplant.
2	An unrelated 6-month-old infant with a congenital (born with) liver disease (biliary atresia) requires a liver transplant.
3	Your adult sibling with alcohol related liver disease requires a liver transplant.
4	An unrelated adult with alcohol related liver disease requires a liver transplant.
5	Your adult sibling with liver disease due to an immune disorder requires a liver transplant.
6	An unrelated adult with liver disease due to an immune disorder requires a liver transplant.

#### 2.5.7 Interpersonal reactivity index (optional)

Participants had the option of completing or skipping the Interpersonal Reactivity Index (16). The Interpersonal Reactivity Index is a 28-item multi-dimensional measure of empathy with four 7-item subscales. Two subscales measure “emotional empathy,” Empathic Concern (EC) and Personal Distress (PD), and two subscales measure “cognitive empathy,” Perspective-Taking (PT) and Fantasy (F). Participants rate each of the 28 statements from 0 (does not describe me well) to 4 (describes me very well). This survey takes approximately 10 min to complete. Responses to each subscale are averaged to obtain an overall score for each sub-dimension of empathy. Reliability ranges (across multiple studies) for each subscale have been found to be as follows: (FS, 0.63 < α < 0.84; PT, 0.65 < α < 0.81; EC, 0.65 < α < 0.82; PD, 0.57 < α < 0.82) (16).

### 2.6 Data analysis

All data were analyzed using SPSS and significance level alpha = 0.05 two-tailed was specified for all tests. Three groups of participants were defined by their answer of either ‘yes,’ ‘maybe,’ or ‘no’ to whether they would be willing to consider donating an organ or part of an organ non-directed (to a stranger). A Kruskal-Wallis test evaluated the association between willingness to consider non-directed donation and scores on the altruism facet of the HEXACO-PI-R.

In the subset who completed the IRI, a one-way ANOVA evaluated association between cumulative IRI scores and willingness to consider non-directed donation (Yes, No, Maybe). One-way ANOVA or Kruskal-Wallis tests (when outcomes were not approximately normally distributed) were used to compare scores for each of the IRI subscales (EC, PD, PT, and F) with willingness to consider donating non-directed.

Average scores for motivating factors and discouraging factors used to identify the 4 most motivating and 4 most discouraging factors, respectively. Kruskal-Wallis tests were used to compare levels of motivation for each of the most motivating and discouraging factors against willingness to consider non-directed donation (Yes, No, Maybe).

Vignettes were combined into categories, including scenarios in which the potential liver recipient was any adult with liver disease (scenarios 4 and 6), any adult with an immune disorder (scenarios 3 and 5), any adult (scenarios 3, 4, 5, and 6), any infant (scenarios 1 and 2), any relative (scenarios 2, 4, and 6), and any nonrelative (scenarios 1, 3, and 5). Scores for these combined categories were the mean scores of all scenarios included in that category. Wilcoxon signed-rank tests were used to compare willingness to donate scores for the following paired scenarios: adult with liver disease and adult with an immune disorder, adult and infant, and relative and nonrelative. A Wilcoxon signed-rank test was also used to compare scenario 4, an unrelated adult with alcohol-related liver disease, with scenario 5, an adult sibling with alcohol-related liver disease.

## 3 Results

### 3.1 Quantitative data

Three hundred-twenty-six participants scanned a QR code to complete the survey, but not all participants responded to every question. Data was collected from July 2, 2024, to July 17, 2024. Most participants identified as female (*N* = 233 of 323, 72%) while 24% identified as male, and 4% identified as non-binary/third gender. Most participants identified themselves as White/Caucasian (*N* = 178 of 323, 55%), 14% identified as Hispanic/Latino, 14% Asian/Pacific Islanders, 4% Black/African American, 0.3% Native American/American Indian, and 12% identified as More than One Race. The average age of participants was 30 years (range of 18–70). Most participants had a bachelor’s degree (*N* = 151 of 323, 46%) or doctoral degree (*N* = 62 of 330, 19%) as their highest level of education. Most participants reported incomes less than $100,000 (*N* = 270 of 322, 84%) (Table 4).

**Table 4:** Demographics of 323 adult survey participants.

	Number of participants	Percent (%)
Gender		
Female	233	72.1
Male	77	23.8
Non-Binary/Third Gender	13	4.0
Race/Ethnicity		
White/Caucasian	178	55.1
Hispanic/Latino	46	14.2
Black/African American	13	4.0
Native American/American Indian	1	0.3
Asian/Pacific Islander	45	13.9
More than One Race	40	12.4
Highest Level of Education		
None	1	0.3
Some High School	2	0.6
High School Diploma or Equivalent	35	10.8
Associate’s Degree	15	4.6
Bachelor’s Degree	151	46.7
Master’s Degree	51	15.8
Doctoral Degree	62	19.2
Trade School	6	1.9
Current Income (*N* = 322)		
Less than $25,000	95	29.5
$25,000–$49,999	65	20.2
$50,000–$99,999	110	34.2
$100,000–$124,999	13	4.0
$125,000–$149,999	9	2.8
$150,000–$174,999	3	0.9
$175,000–$200,000	3	0.9
More than $200,000	24	7.5

**Figure 1:**
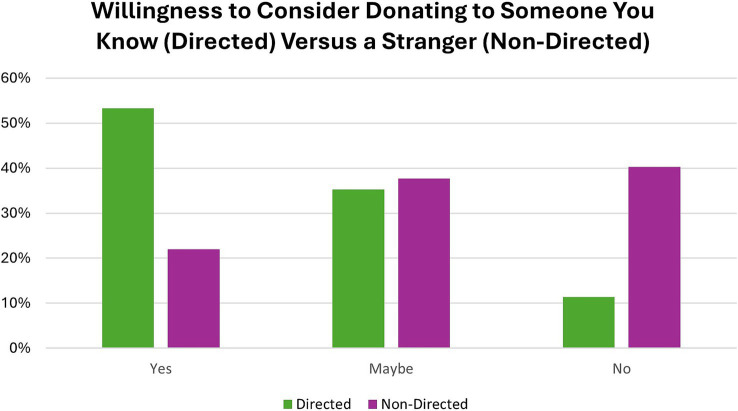
Participants were asked whether they would be willing to donate part of their liver or a kidney to someone they knew (directed) and/or a stranger (non-directed).

Most participants were aware of both living kidney and living liver donation (*N* = 233 of 318, 73%). Six percent (*N* = 19 of 318) of participants were not aware of either living kidney or living liver donation, 16% (*N* = 51 of 318) were only aware of living kidney donation, and 5% (*N* = 15 of 318) were only aware of living liver donation. Of those with any awareness of living donation (kidney and/or liver) (*N* = 299), one participant had donated a kidney. More than half of participants said that they would be willing to donate a kidney and/or part of their liver to someone they knew (*N* = 169 of 317, 53%). Thirty-five percent (*N* = 112 of 317) of participants said maybe they would donate, and 11% (*N* = 36 of 317) said they would not donate to someone they knew (Figure 1).

**Figure 2:**
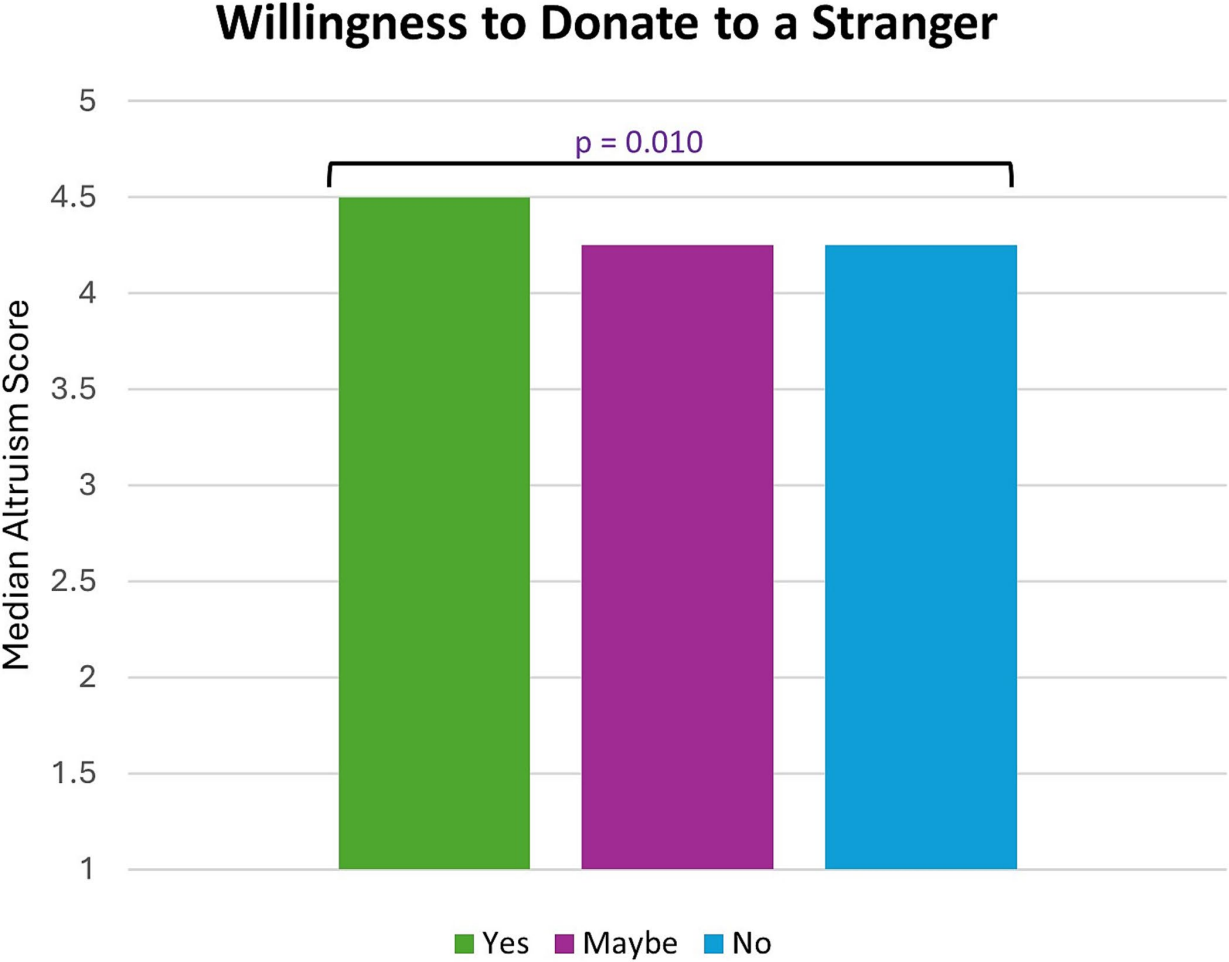
Participants who were willing to donate to a stranger had the highest median altruism score (*p*-value determined by Kruskal-Wallis test).

Most participants were also aware of non-directed living donation (*N* = 245 of 305, 80%). Only 22% of participants said, yes, they would consider non-directed living donation (*N* = 67 of 305), while 38% (*N* = 115 of 305) said maybe, and 40% (*N* = 123 of 305) said they would not consider non-directed donation (Figure 1). Participants higher in altruism were more likely to endorse considering non-directed donation [*H* (2) = 9.291, *p* = 0.010] (Figure 2).

Participants who elected to fill out optional IRI questions (*N* = 68, 22%) had significantly higher altruism scores (z = −3.912, *p* < 0.001) than those who did not answer the questions. No statistically significant relationships were observed between IRI scores and willingness to consider non-directed donation [*F* (2,61) = 2.350, *p* = 0.104].

**Figure 3:**
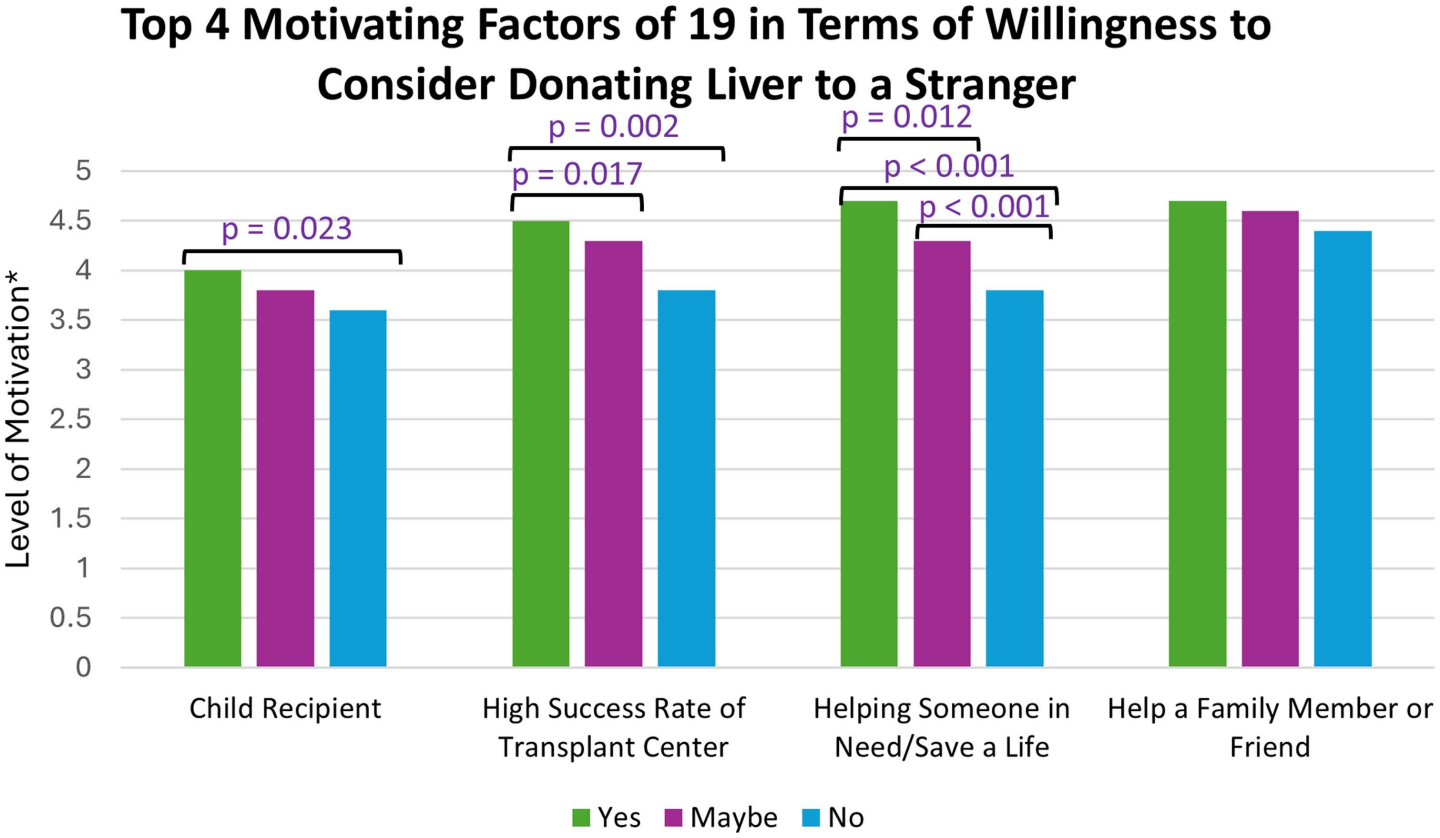
*(1 = not at all motivating and 5 = extremely motivating); The top four motivating factors were compared against participants’ willingness to consider non-directed donation. *p*-values were determined by a Kruskal-Wallis test.

The most motivating factors (on a scale of 1–5, with 5 being the most motivating) for considering living donation included having a child recipient (mean score of 3.77), helping someone in need and potentially saving a life (mean of 4.13), a high transplant center success rate (mean of 4.14), and helping a family member or friend (mean of 4.56). Participants who answered yes to considering non-directed donation had statistically significant higher levels of motivation than participants who answered no for the following factors: having a child recipient [*H* (2) = 7.577, *p* = 0.023], helping someone in need and potentially saving a life [*H* (2) = 46.722, *p* < 0.001], and a high transplant center success rate [*H* (2) = 12.312, *p* = 0.002] (Figure 3).

**Figure 4:**
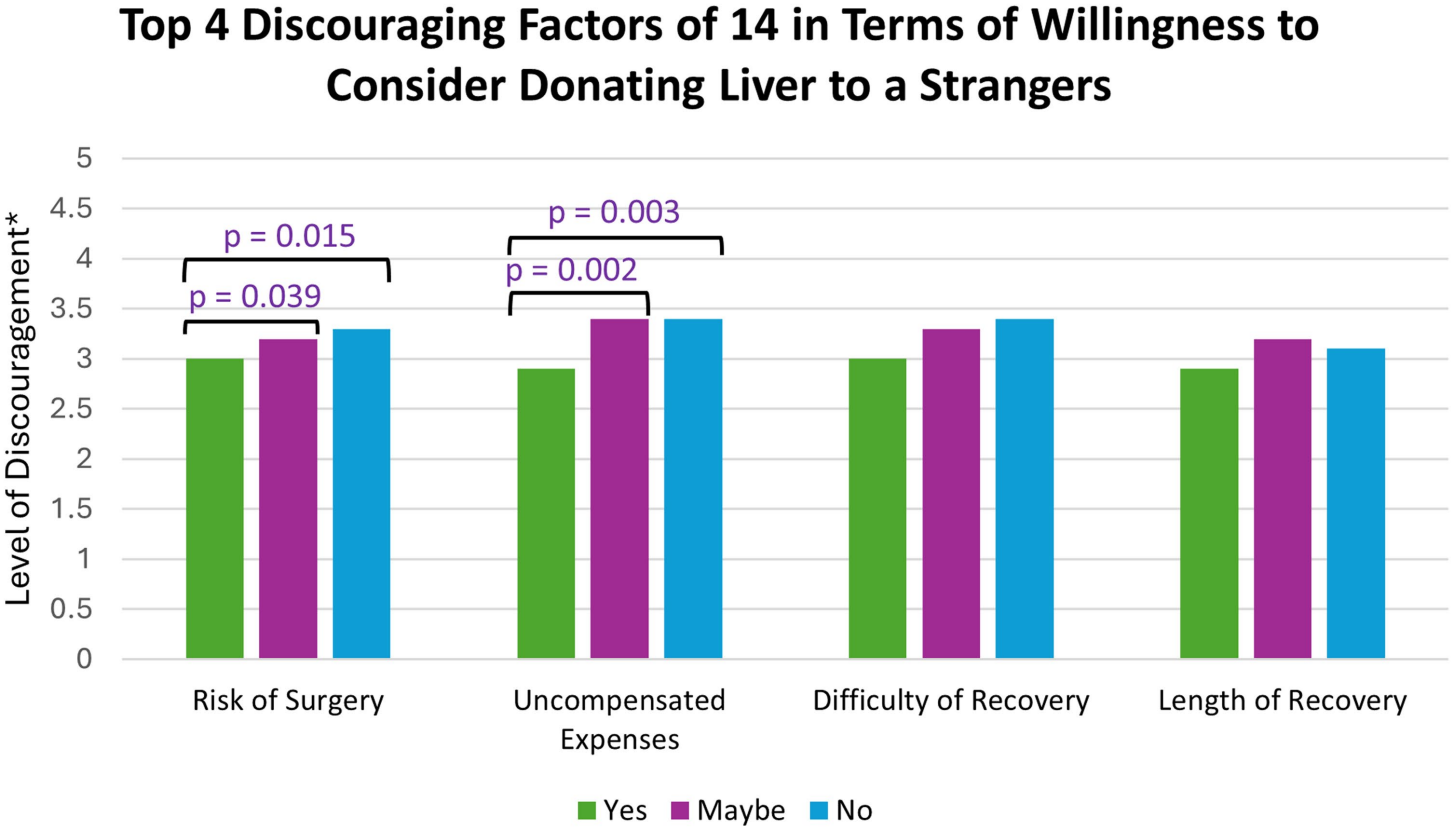
*(1 = not at all discouraging and 5 = extremely discouraging); The top four discouraging factors were compared against participants’ willingness to consider non-directed donation. *p*-values were determined by a Kruskal-Wallis test.

The most discouraging factors (on a scale of 1–5, with 5 being the most discouraging) for considering living donation included uncompensated expenses related to the surgery (mean score of 3.26), difficulty of surgery recovery (mean of 3.26), risk of surgery (mean score of 3.21), and length of surgery recovery (mean of 3.07). Participants who answered yes to considering non-directed donation had statistically significant lower levels of discouragement than participants who answered no for uncompensated expenses related to surgery [*H* (2) = 11.409, *p* = 0.003] and risk of surgery [*H* (2) = 8.391, *p* = 0.015] (Figure 4).

**Figure 5:**
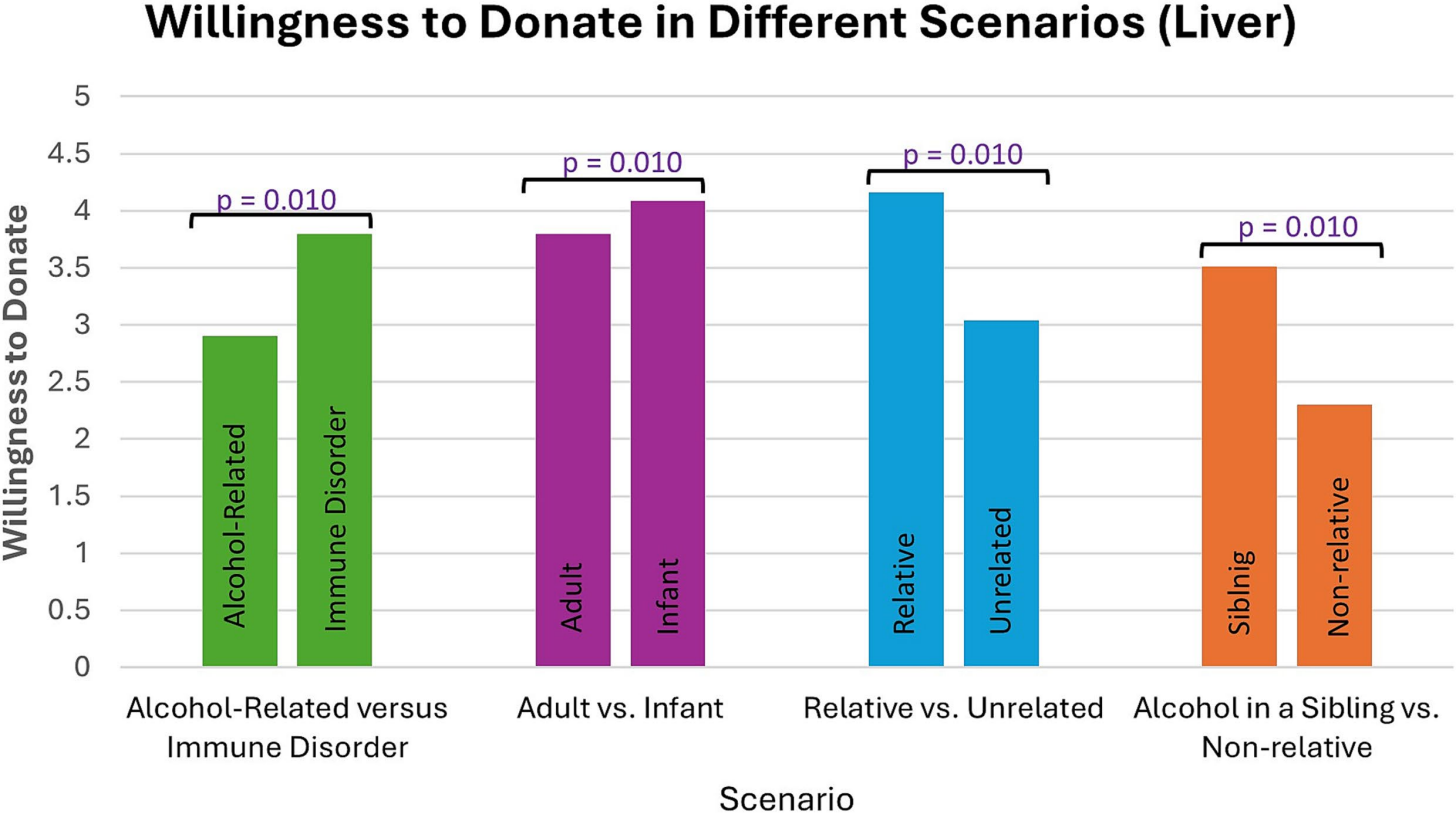
Participants were more willing to consider donating part of their liver in some scenarios compared to others. *p*-values were determined by a Wilcoxon signed-rank test.

Participants were more willing to consider donating a part of their liver to a recipient with an immune disorder than a recipient with alcohol-related liver disease (z = −11.959, *p* < 0.001), an infant recipient rather than an adult recipient (z = −6.740, *p* < 0.001), a relative rather than a nonrelative (z = −13.381, *p* < 0.001), and a sibling with alcohol-related liver disease rather than a nonrelative with alcohol-related liver disease (z = −11.929, *p* < 0.001) (Figure 5).

Participants who elected to fill out optional IRI questions (*N* = 68, 22%) had significantly higher altruism scores (z = −3.912, *p* < 0.001) than those who did not answer the questions. No statistically significant relationships were observed between IRI scores and willingness to consider non-directed donation [*F* (2,61) = 2.350, *p* = 0.104].

### 3.2 Qualitative responses

Participants were asked why they answered either “yes,” “maybe,” or “no” to considering non-directed donation (NDD). Some participants who answered “yes” to considering NDD expressed altruistic or empathetic reasons for considering donating. A few participants expressed that they would prefer to donate anonymously, with one participant saying, *“It is sometimes better to keep things professional and clinical.”* There were far more participants expressing the opposite statement, that they would not consider NDD without a close relationship with the recipient. Another concern with donating to a stranger was that they may “waste” the organ, for example if the disease requiring transplant is “self-inflicted” due to alcohol or another lifestyle choice. A common concern was the financial impact from the surgery. Participants also expressed concerns over the risk of the surgery and potential changes to their health (Table 5).

**Table 5:** Qualitative response.

Please explain why you would or would not consider donating to an anonymous recipient:
**Reasons given for or when considering NDD**
*Would love to donate to help people and save people!*
*Everyone’s life matters and not everyone has family/friends that would be a match.*
*I would choose to donate because I think about my family who is in need and I would want the same.*
*As part of transplant chain that would enable a loved one to get a transplant if I could not donate directly to them*
*I would consider doing an anonymous donation if the recovery and cost to me was minimal.*
*I think I would consider it if someone really needed it and I could donate and the opportunity presented itself*
*[Considering the] checks and balances in place…I would be willing to donate to anyone who has been approved and in need of an organ, even not knowing them.*
**Concerns about NDD**
*[I] would not be able to see the impact.*
*It just feels more disconnected…*
*it would be a hard process to do for someone I do not know.*
*What if a family member needs one day and I do not have it anymore?*
*It’s taboo for me to donate organs and blood…due to cultural reasons.*
*I would want to know that it would not be ‘immediately wasted’ and thus not really have as much impact*
*I need to know what is wrong with them. Alcohol is self-inflicted for example.*
*If I did not know the person, I’d be more reluctant because I do not know if the person would take care of the transplant*
*… I cannot afford to not be compensated for time and expenses related to the procedure…*
*I think the risk of harm during surgery to myself is [too] big of a factor to overcome when considering this situation.*
*The high risks of surgery make me very hesitant.*
*I am most worried about the possibility that I’d need the liver or kidney later and that I’d be less healthy without an extra liver or kidney. I do not know if this is true.*
*…As someone that does not have insurance, this is a deterrent for me, but I would be willing to sacrifice for my immediate family members.*

Participants had the option to explain why they would or would not consider non-directed donation (NDD). Examples from 136 qualitative responses are included in this table.

## 4 Discussion

The findings from this study have the potential to shape future educational and outreach interventions about living organ donation in the United States. Living organ donation has the potential to save thousands of lives and reduce lengthy waitlist times for patients in end-stage organ failure. In the US, there remains a shortage of individuals motivated to become living donors, and even fewer individuals pursuing living liver donation. This study provides insight into the motivating and discouraging factors for individuals considering living donation as well as donor preference for directed donation versus non-directed donation.

Among those interested in living donation, the top four motivating factors identified were: (1) donation to a child, (2) helping a friend or family member, (3) helping someone in need or saving a life, and (4) high success rates of the transplant center. Willingness to consider living organ donation was high, with individuals being more likely to consider pursuing directed donation (53%) over non-directed donation (22%).

A novel contribution of this study is measuring the interaction between willingness to donate and the prospective recipient’s etiology of disease. Individuals are also more likely to pursue liver donation to a child, relative, or to an adult with non-alcoholic liver disease such as an autoimmune disorder. This is despite alcohol-related liver disease being the most common indication for liver transplant in the US (17). Poor understanding of or even stigma about alcohol use disorder and alcohol-related liver disease could be contributing factors to the scarcity of living liver donors (18). Public stigma about alcohol-related liver disease is well documented in the literature and involves viewing alcohol use disorder as a self-inflicted moral problem rather than a medical illness requiring treatment (19, 20). Proper assessment of alcohol use disorder and completion of substance abuse treatment is often required pre-transplant for individuals with alcohol-related liver disease (21). For individuals considering living liver donation, they may be unaware of this comprehensive evaluation process, or the steps taken to mitigate future alcohol relapse risk with alcohol use disorder/alcohol-related liver disease.

This study suggests that when alcohol-related liver disease is present, individuals are more likely to donate to a sibling or relative rather than a stranger, though willingness to donate to a non-relative with alcohol-related liver disease is still relatively high (average willingness score = 2.31 on a scale of 1 to 5, with 1 being “very unwilling to donate” and 5 being “very willing to donate”). There are several possible reasons for this preference. It is possible that direct observation of end-stage liver disease or seeing the physical symptoms of liver disease (e.g., jaundice, ascites, fatigue, confusion) could increase donor motivation amongst relatives. In the authors’ clinical experience, directed donors often express emotional discomfort with seeing the health decline of someone they know. Additionally, it is possible that having direct knowledge about the intended recipient’s alcohol history or phase of recovery could influence donor decision making (22). Additional research is needed about living liver donors and their views about addiction, recovery, and treatment effectiveness.

Other factors contributing to pursuing living donation include personality features seen amongst the donor population, such as greater drive to help someone in need or a desire to save a life, as well as higher rates of altruism particularly for individuals considering non-directed donation versus directed donation (5). Of note, those pursuing non-directed donation have little to no information about their intended recipient. Thus, it is possible that motivation to pursue NDD is separate and unassociated from one’s knowledge of liver disease etiology. Additional research about NDD is encouraged to better understand this relationship. Lastly, the success rate of the transplant center was also identified as a motivating factor, suggesting that perceived trust in one’s transplant team or the center motivates donors to proceed with elective donor surgery.

The top four discouraging factors for both directed and non-directed donation were: (1) perceived surgical risk, (2) difficulty of surgical recovery, (3) length of recovery, and (4) financial cost or uncovered expenses. When compared to kidney donation surgery, living liver donors tend to have longer hospital stays and longer recovery periods (23–25). Engagement in preventative healthcare visits and health behavior changes with nutrition, physical activity, and alcohol moderation are also strongly encouraged post-donation surgery; liver donors are also asked to abstain from alcohol for 6 months to one-year after surgery which can be challenging for some (26, 27). Consistent with the literature, additional socioeconomic circumstances can hinder one’s motivation to donate such as unexpected financial burden, lack of health insurance coverage, or loss of wages (10).

This study is limited by its relatively small sample size and homogenous participant population. Of the 348 individuals surveyed, the majority identified as White/Caucasian, female, and completed at least trade school education or greater. This study may not represent the attitudes of other racial or ethnic groups or genders other than female. The study was conducted on an academic medical campus, which may introduce selection bias. For example, individuals may be more likely to hold positive beliefs or attitudes about health-related behavior. Further, these individuals may be more aware of living donation than the general population. This could lead to overestimation of positive views towards and the willingness to consider living donation. Next steps are to distribute surveys to other populations outside a medical campus to determine if this affects views on living organ donation. Future studies could explore additional motivating and discouraging factors, such as religious beliefs, personal health status, or exposure to social media. This study is cross-sectional, and longitudinal data will be necessary to understand the impact of any educational campaigns or other interventions.

## Data Availability

The raw data supporting the conclusions of this article will be made available by the authors, without undue reservation.
